# Circulating miR-208b and miR-34a Are Associated with Left Ventricular Remodeling after Acute Myocardial Infarction

**DOI:** 10.3390/ijms15045774

**Published:** 2014-04-04

**Authors:** Pin Lv, Mingxia Zhou, Jing He, Weiwei Meng, Xuehan Ma, Shuling Dong, Xianchun Meng, Xue Zhao, Xi Wang, Fucheng He

**Affiliations:** 1 Department of Medical Laboratory, the First Affiliated Hospital, Zhengzhou University, Zhengzhou 450052, China; E-Mails: lvpinkl555@gmail.com (P.L.); mengweiwei23@outlook.com (W.M.); lpmaxuehan@sina.com (X.Ma); zzhdongshuling@126.com (S.D.); mengxianchun3@sina.com (X.Me.); zhaoxue86@outlook.com (X.Z.); 2 Institute of Clinical Medicine, the First Affiliated Hospital, Zhengzhou University, Zhengzhou 450052, China; 3 Basic Medical College, Zhengzhou University, Zhengzhou 450052, China; E-Mails: lpzhoumingxia@outlook.com (M.Z.); lphejing@sina.com (J.H.); 4 Department of Cardiology, the First Affiliated Hospital, Zhengzhou University, Zhengzhou 450052, China; E-Mail: wangxilp@outlook.com

**Keywords:** microRNAs, myocardial infarction, left ventricular remodeling, prognosis, circulating biomarkers

## Abstract

Left ventricular remodeling after acute myocardial infarction (AMI) is associated with adverse prognosis. It is becoming increasingly clear that circulating miRNAs could be promising biomarkers for various pathological processes in the heart, including myocardial infarction, myocardial remodeling and progression to heart failure. In the present study, a total of 359 consecutive patients were recruited. Plasma samples were collected on admission. Echocardiographic studies were performed during the admission and at six months follow-up after AMI. Remodeling was defined as an at least 10% increase from baseline in the left ventricular end-diastolic volume. Plasma miRNA levels were assessed for association with six months mortality or development of heart failure. Results showed that levels of plasma miR-208b and miR-34a were significantly higher in patients with remodeling than those without. Increased miRNA levels were strongly associated with increased risk of mortality or heart failure within six months for miR-208b (OR 17.91, 95% confidence interval = 2.07–98.81, *p* = 0.003), miR-34a (OR 4.18, 95% confidence interval = 1.36–12.83, *p* = 0.012) and combination of the two miRNAs (OR 18.73, 95% confidence interval = 1.96–101.23, *p* = 0.000). The two miRNA panels reclassified a significant proportion of patients with a net reclassification improvement of 11.7% (*p* = 0.025) and an integrated discrimination improvement of 7.7% (*p* = 0.002). These results demonstrated that circulating miR-208b and miR-34a could be useful biomarkers for predicting left ventricular remodeling after AMI, and the miRNA levels are associated with increased risk of mortality or heart failure.

## 1. Introduction

Left ventricular (LV) remodeling after acute myocardial infarction (AMI) remains a pivotal clinical issue, despite the advance of medical treatment over the past few decades. Long-term remodeling is associated with increased risk of cardiovascular death and heart failure [1–3]. Therefore, Early prediction of LV remodeling and the development of heart failure in post-AMI patients is needed and may, potentially, improve by the identification of novel biomarkers. To date, several circulating biomarkers have been shown to predict cardiovascular events after AMI, such as *N*-terminal pro-brain natriuretic peptide (NT-proBNP) and cardiac troponins (cTns) [4–6]. However, early studies have reported that the prognostic value of these classical cardiac biomarkers is limited, due to the fact that circulating levels fluctuate considerably in the early period after AMI and are easily influenced by hepatorenal function [7–9]. In recent years, advances in molecular biology and technology have initiated huge interest in nucleotide-based biomarkers that may enhance diagnostic or prognostic effectiveness. MicroRNAs (miRNAs) are a relatively novel class of endogenous, non-coding single-stranded small RNAs that can regulate gene expression at the post-transcriptional level and play critical roles in various pathological and biological processes, including proliferation, cell differentiation, apoptosis, cardiovascular diseases and cancers [10–13]. The fact that most miRNA species are remarkably stable and readily detectable in the peripheral blood or plasma, and that the levels of circulating miRNAs are characteristically altered in individuals with diverse pathological conditions, make them excellent candidate diagnostic and prognostic biomarkers for various diseases [14–17]. Several studies have reported the diagnostic value of circulating miRNAs in the setting of AMI [18–21]. Few reports, however, have examined the predictive value of circulating miRNAs in cardiac remodeling after AMI.

Previous studies have demonstrated that cardiac-specific miR-208b could serve as useful biomarker for early diagnosis of AMI [20,22,23]. Moreover, recent study showed a relationship between plasma miR-208b and LV dysfunction after MI [20]. MiR-34a was demonstrated as a pro-apoptotic factor in cardiac contractile function during ageing and after AMI [24,25]. Therapeutic potential by silencing the miR-34 family in protecting the heart against pathological cardiac remodeling and improving heart function has also been demonstrated in preclinical mouse models [26,27]. Meanwhile, overexpression of miR-34a induced endothelial cells senescence, which plays an important role in atherosclerosis [28]. These results suggested that miRNAs may have diagnostic and therapeutic roles in myocardial diseases. However, the predictive value of circulating miRNAs in cardiac remodeling after AMI have received less attention.

Therefore, the present study aimed to evaluate whether circulating miR-208b and miR-34a expressed after AMI could serve as predictors for LV remodeling. Additionally, we assessed the relationship between miRNA levels and six months mortality or development of heart failure after AMI.

## 2. Results

### 2.1. Baseline Clinical Characteristics of the Study Population

Basic clinical characteristics such as age, gender, total cholesterol, triglyceride, HDL, LDL, systolic blood pressure, diastolic blood pressure, diabetes, smoking history and medication were drawn into the present study. Clinical characteristics of all the patients and divided by evidence of LV remodeling, and by experience of endpoint are summarized in Table 1. The concentrations of cTnT and NT-proBNP on admission, LVEDV and LVEF at follow-up, ΔLVEDV, ΔLVESV and ΔLVEF levels were significantly higher in patients with remodeling than those without (*p* < 0.05). There were no significant differences in any other clinical characteristics between the remodeling and non-remodeling group. NT-proBNP levels on admission, ΔLVEDV, ΔLVESV and ΔLVEF levels were significantly higher in patients in the experienced endpoint group than those in the no endpoint group (*p* < 0.05). There were no significant differences in any other clinical characteristics between the experienced endpoint and no endpoint group.

### 2.2. Circulating miRNA Levels Reflect LV Remodeling after AMI

**Figure 1.**
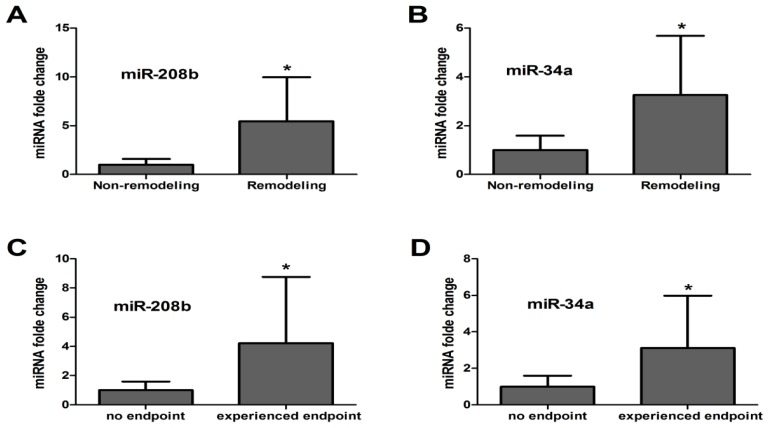
Plasma miRNA-208b and 34a are increased in the remodeling group and experienced endpoint group. Plasma samples were collected at a median of six (range 2–10) h after AMI onset. (**A**,**B**) Mean fold change of plasma miRNA levels between remodeling (*n* = 116) and non-remodeling group (*n* = 243), setting 1 as an arbitrary value for non-remodeling group; (**C**,**D**) Mean fold change of plasma miRNA levels between experienced endpoint (*n* = 83) and no endpoint group (*n* = 276), setting 1 as an arbitrary value for no endpoint group; Independent-samples *T* test was used for two-group comparisons. Results were reported as mean ± SD (* *p* < 0.05).

To assess the value of plasma miR-208b and miR-34a for predicting LV remodeling after AMI, we categorized all the patients into remodeling (*n* = 116) and non-remodeling (*n* = 243). LV remodeling was defined as at least 10% increase from the baseline in LVEDV during follow-up. Plasma miRNA levels were determined using qRT-PCR. The results were summarized in Table 2. Independent-samples *T* test showed statistical differences of plasma miR-208b and miR-34a levels between remodeling and non-remodeling group. Results of 2^−ΔΔ^*^C^*^t^ analysis showed that plasma concentrations of miR-208b and miR-34a were both markedly elevated in patients with remodeling relative to those without, fold 4.81 (±3.57) and 3.26 (±2.42) respectively (Figure 1A,B).

### 2.3. Circulating miRNAs as Potential Predictors of LV Remodeling after AMI

**Figure 2.**
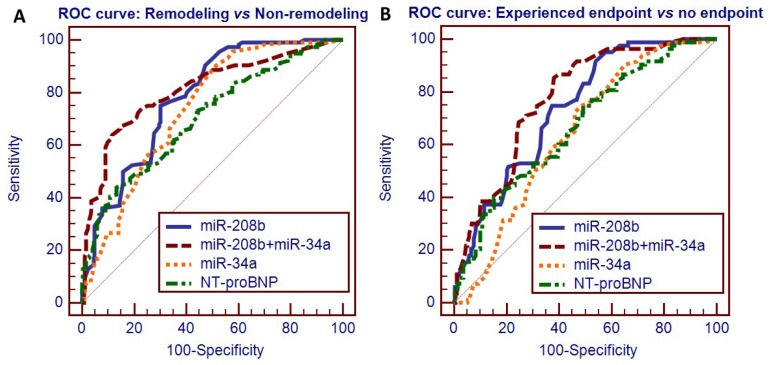
Plasma miRNA levels associated with prognosis after AMI. (**A**) Receiver operating characteristic (ROC) curve for plasma miR-208b, miR-34a and NT-proBNP discriminate remodeling from non-remodeling group; (**B**) ROC curve for plasma miR-208b, miR-34a and NT-proBNP discriminate experienced endpoint from no endpoint group.

ROC curve analysis was performed to evaluate the predictive power of plasma miR-208b and miR-34a for LV remodeling after AMI. The ability to discriminate the patients with remodeling from those without was determined according to the AUC of 0.780 (95% confidence interval, 0.734–0.822; *p* < 0.001) for miR-208b and 0.738 (95% confidence interval, 0.689–0.783; *p* < 0.001) for miR-34a, respectively. The combination of miR-208b and miR-34a showed an AUC of 0.812 (95% confidence interval, 0.767–0.851; *p* < 0.001). The AUC measured for NT-proBNP was 0.704 (95% confidence interval, 0.654–0.751; *p* < 0.001) (Figure 2A). The comparison of ROC curves between miR-208b and Nt-proBNP was of borderline statistical significance (*z =* 1.96, *p* = 0.05). MiR-34a was not superior to NT-proBNP in the prediction (*z* = 0.875, *p* = 0.381). However, the combination of the two miRNAs showed a superior predictive power compared to Nt-proBNP (*z* = 2.605, *p* = 0.009).

### 2.4. Prognostic Value of Circulating miRNAs after AMI

Cardiogenic death or development of heart failure during follow-up was considered as primary endpoint. A total of 83 patients (23.1%) experienced the primary endpoint. As shown in Figure 1C,D, levels of miR-208b and miR-34a were both elevated in patients who experienced primary endpoint, compared to those with no endpoint (*p* < 0.05), fold 4.21 (±4.55) and 3.12 (±2.86) respectively. The ability of plasma miR-208b, miR-34a and combined miRNAs to discriminate the experienced endpoint group from those with no endpoint was evaluated by the ROC curve with an AUC of 0.737 (95% confidence interval, 0.689–0.782; *p* < 0.001), 0.642 (95% confidence interval, 0.590–0.691; *p* < 0.001) and 0.777 (95% confidence interval, 0.731–0.819; *p* < 0.001), respectively. The AUC measured for NT-proBNP was 0.669 (95% confidence interval, 0.617–0.717; *p* < 0.001) (Figure 2B). Comparison of ROC curves showed that neither of the single miRNAs was superior to NT-proBNP in predicting endpoint after AMI (miR-208b *vs.* NT-proBNP: *z* = 1.526, *p* = 0.127; miR-34a *vs.* NT-proBNP: *z* = 0.629, *p* = 0.529). While the combined miRNAs showed a higher predictive power than NT-proBNP (*z* = 2.496, *p* = 0.013). Multivariate logistic regression analysis showed that Odds Ratios (95% confidence interval), adjusted for age, gender, current smoking, cTnT, NT-proBNP, and time from AMI onset to sampling were 17.91 (2.07–98.81, *p* = 0.003) for miR-208b, 4.18 (1.36–12.83, *p* = 0.012) for miR-34a and 18.73 (1.96–101.23, *p* = 0.000) for the combination of miR-208b and miR-34a, respectively.

### 2.5. Reclassification Analyses for the Circulating miRNAs in Predicting LV Remodeling after AMI

Reclassification analyses were performed to evaluate the added value of each miRNA and both miRNAs over a multi-parameter clinical model including age, gender, current smoking, cTnT, NT-proBNP, and time from AMI onset to sampling. Patients were categorized into low (<10%), intermediate (10%–30%) and high (>30%) probability of remodeling groups. Results showed that the capacity to reclassify patients originally misclassified by the multi-parameter clinical model into remodeling and no remodeling was 9.5% (NRI = 0.095, *p* = 0.039) for miR-208b, 6.5% (NRI = 0.065, *p* = 0.177) for miR-34s and 11.7% (NRI = 0.117, *p* = 0.025) for the combination of miRNAs, respectively (Tables 3–5). Again, the IDI calculated for miR-208b, miR-34a and the combination of miRNAs was 0.042 (*p* = 0.013), 0.008 (*p* = 0.150) and 0.077 (*p* = 0.002), respectively.

## 3. Discussion

Acute myocardial infarction (AMI) commonly triggers left ventricular (LV) remodeling, which has a powerful association with adverse outcome, involves progressive deterioration of cardiac function and development of heart failure [3,29]. Therefore, early identification of this consequence of AMI is clinically very important. Recent studies demonstrate that miRNAs can be exported or released by cells and circulate in bloodstream [15,30]. Tissue and disease specificity [31,32], rapid release dynamics and stability in circulation make miRNAs promising candidates for diagnostic biomarkers in a wide range of disease states [15], especially in the cardiovascular disease [30]. However, their prognostic utility has been little studied.

The present study has led us to determine that circulating miRNAs can be clinically prognostic biomarkers for patients who suffer from AMI. In the plasma analysis, we found that concentrations of miR-208b and miR34a on admission were significantly elevated in patients with LV remodeling compared to those without. ROC analysis, and multivariate logistic regression further indicated the two miRNAs might be good biomarkers for AMI prognosis. Comparison of the ROC curves showed that the combination of miR-208b and miR-34a outperformed NT-proBNP in both LV remodeling and experienced endpoint prediction after AMI. Odds Ratios from multivariate logistic regression, adjusted for age, gender, current smoking, cTnT, NT-proBNP and time from AMI onset to sampling were 17.91 for miR-208b, 4.18 for miR-34a and 18.73 for the combination of the two miRNAs, respectively. Since the diagnostic potential of circulating miRNAs in the setting of AMI has been demonstrated both in human and mouse models [18–21], our present research provides evidence that plasma miRNAs are associated with outcomes post AMI that can be used for prognostication purposes. Moreover, our results were consistently with previous studies showing that high levels of miR-208b are associated with LV dysfunction after AMI [20], and serum levels of miR-34a elevated 18 days after AMI onset could serve as a predictive indicator of heart failure in patients [33]. In the reports published by Matsumoto *et al*. [33], activation of p53 and the increased expression of the p53-responsive microRNAs, miR-192, miR-194, and miR-34a, are likely involved in the pathogenesis of HF after AMI. Although we have identified the dysregulation of miR-208b and 34a may help predicting outcome post AMI, further experimental studies are needed to explore the mechanism(s) of the dysregulation since the levels of circulating miRNAs may be affected by multiple parameters such as the change in expression in the tissue, the release of the miRNAs by cells into the circulation and the stability of miRNAs in plasma.

Considering the prognostic value of circulating miRNAs after MI, Zile *et al.* [34] found that a specific temporal changes in plasma miRNAs occur in patients during the LV remodeling process that follows a myocardial infarction (post-MI). Another report published by Widera *et al.* [35] showed that levels of plasma miR-133a and miR-208b on admission could reflect the risk of death in AMI patients upon adjustment for age and gender. Matsumoto *et al.* [36] reported that serum levels of miR-155 and miR-380 * at the time of discharge after AMI were higher in patients who subsequently experienced cardiac death within 1 year. Again, in a study by Devaux *et al*. [37], low circulating levels of miR-150 are associated with LV remodeling after the first ST-AMI. In addition to these, several other circulating miRNAs were also involved in cardiac remodeling after MI, such as miR-16, 27a and 101 *etc.* [38]. These results support our stance that circulating miRNAs could serve as available biomarkers to reflect cardiac remodeling and outcome after AMI. Moreover, in our research, combination of plasma miR-208b and miR-34a outperformed NT-proBNP in both LV remodeling and experienced endpoint prediction after AMI, and the two miRNA panels could reclassify 11.7% of patients misclassified by a multi-parameter clinical model including age, gender, current smoking, cTnT, NT-proBNP, and time from AMI onset to sampling. These results suggest that instead of seeking one gold standard biomarker, a combination of multiple biomarkers strategy can provide more information in the prognosis. However, we must consider that many patients are not available in the very early period after AMI. In our study, higher plasma levels of miR-208b and miR-34a, assayed at the median of 6 h after AMI onset, could serve as predictors of LV remodeling and associated with 6 months outcome in patients. Despite a modest prognostic power, our present study lays the groundwork for future efforts to identify and develop miR-208b and miR-34a (perhaps also other miRNAs) as novel class of blood-based biomarkers for AMI prognosis.

Several limitations of this study should be acknowledged. First, the consideration of circulating miR-208b and miR-34a as biomarkers for AMI prognosis is at present based on our results from a relatively small sample size and larger clinical studies are definitely required to support our results; Second, we only measured miRNA concentrations on admission; measurement of miRNA levels at follow up might give additional information on clinical outcome; Finally, the technology used to detect miRNAs requires optimization, and further studies are needed to establish a standardized data normalization method for obtaining accurate and reproducible results.

In conclusion, the results of our study revealed that plasma miR-208b and miR-34a could serve as available predictors for LV remodeling after AMI and were associated with the six months mortality or development of heart failure.

## 4. Experimental Section

### 4.1. Participants

Three hundred and fifty-nine consecutive patients diagnosed with AMI were enrolled in this study from the First Affiliated Hospital of Zhengzhou University (Zhengzhou, China) between December 2011 and October 2012. Patient characteristics are described in Table 1. Diagnosis of AMI was based on combination of several parameters: (1) ischemic symptoms; (2) increased levels of myocardial necrosis markers (troponins (cTns) and creatine kinase (CK)) to above twice the upper limit of the normal range; (3) pathological Q wave; and (4) ST-segment elevation or depression [39]. Patients with previous MI or PCI, hematological diseases, acute or chronic infection, significant renal or hepatic dysfunction, and known or treated malignancies were excluded. Hypertension was defined according to the presence of elevated systolic (>140 mmHg) and/or diastolic (>90 mmHg) blood pressure or the current use of antihypertensive drugs. Definition of diabetes mellitus was based on clinical features and requirement of dietary treatment and/or medical therapy to control blood glucose levels. Hyperlipidaemia was defined as serum total cholesterol levels ≥5.2 mmol/L, or triglycerides ≥1.7 mmol/L, or low density cholesterol ≥2.6 mmol/L, or use of statin medication [40]. Patient was considered as a smoker if he/she was smoking at the current moment or was a smoker in the past.

Echocardiographic studies were completed by three cardiologists. However, the same operator analyzed the same patient during the hospitalization (approximately 1–4 days after admission) and at the six-month follow-up. Patients were categorized according to whether they demonstrated LV remodeling post AMI, as assessed from the change (ΔLVEDV) in LVEDV between admission and follow-up. LV remodeling was defined as at least 10% increase from the baseline in LVEDV during follow-up [41]. For the assessment of the prognostic value of circulating miRNA levels, the cardiogenic death (*n* = 19) or development of heart failure (clinical diagnosis (*n* = 25), an ejection fraction <40% (*n* = 61)) during follow-up was considered as primary endpoint. Subjects were classified as heart failure when they met the Framingham criteria for the clinical diagnosis, and if circulating NT-proBNP was above the age-related cutoff points published by Januzzi *et al*. [42].

### 4.2. Plasma Collection and Storage

Fasting venous blood samples from AMI patients were collected in tubes containing EDTA—K_2_ on admission (a median of 6 (range 2–10) h after the onset of symptoms). Samples were centrifuged at 3000× *g* for 10 min at 4 °C, then the supernatant was isolated and centrifuged at 12,000× *g* for 10 min at 4 °C. Plasma was collected and stored in aliquots at −80 °C until analysis.

### 4.3. RNA Preparation

Two hundred microliters of plasma were spiked with miScript miRNA mimic SV40 (Qiagen, Hilden, Germany, 2 μM, 1 μL per 100 μL plasma). Total RNA was extracted from these plasma samples using TRI Reagent BD (MRC, TB-126, Cincinnati, OH, USA) according to the manufacturer’s protocol and dissolved in 10 μL diethylpyrocarbonate (DEPC)-treated water. The concentration and quality of the RNA samples was determined using NanoDrop spectrophotometer (NanoDrop, Thermo Fisher Scientific, Waltham, MA, USA).

### 4.4. MiRNA Determination

Total RNA (0.5 μg) from each sample was reverse transcribed using miRNA-specific stem-loop RT primer. The PrimeScript^OeR^ RT reagent Kit With gDNA Eraser (TaKaRa, DRR047S, Tokyo, Japan) was used according to the kit procedures. In brief, the 20 μL reactions were incubated for 15 min at 42 °C, followed by 5 s at 85 °C, and the resulting cDNA was stored at −20 °C for the following quantitative real-time PCR. qRT-PCR was performed on ABI 7500 Fast Real-Time PCR System (Applied Biosystems, Foster City, CA, USA) using SYBR^OeR^ Premix Ex Taq™ II (TaKaRa, DRR820S, Tokyo, Japan) according to the manufacturer’s instructions. Briefly, the thermal cycling consisted of an initial denaturation at 95 °C for 30 s, followed by 40 cycles of 95 °C for 3 s and 60 °C for 30 s. A melt curve was performed after each reaction. All PCR reactions yielded a single peak on the melt curve, indicating acceptable specificity of the primers. Assays were performed in triplicate. Resultant miRNA levels were normalized using spiked-in SV40. Data were analyzed with 7500 Fast System SDS Software version 1.4.0.25 (Applied Biosystems, Foster City, CA, USA) with the automatic *C*_t_ setting for assigning baseline and threshold for *C*_t_ determination. The *C*_t_ values greater than 36 were considered as not expressed. MiRNA relative expression levels were calculated by the equation of 2^−Δ^*^C^*^t^, (Δ*C*_t_ = *C*_t target_ − *C*_t spiked-in SV40_). Fold change in miRNA levels were calculated using the 2^−ΔΔ^*^C^*^t^ method [43].

### 4.5. Statistical Analysis

SPSS Statistics software version 20.0 (IBM, Armonk, NY, USA) was used for the statistical analysis. All data were subjected to a normality test (Kolmogorov-Smirnov). Continuous data are presented as mean ± SD or median with interquartile range. Categorical variables are presented as counts and percentage. Independent-samples *t*-test and Mann-Whitney *U* test were used to compare two groups of continuous variables and Chi-Square test for categorical variables. Receiver operating characteristic (ROC) curve analysis and the comparision of the derived area under the curve (AUC) were performed using Medcalc software version 13.0.2 to estimate the predictive power of biomarkers. Prediction of miRNA levels for cardiogenic death or development of heart failure was assessed by multivariate logistic regression. Reclassification analyses were performed, evaluating the predictive value for the identification of remodeling of each miRNA and both miRNAs together with a multi-parameter clinical model including age, gender, current smoking, cTnT, NT-proBNP, and time from AMI onset to sampling. Logistic regression models were used for patient classification. The net reclassification index (NRI) and the integrated discrimination improvement (IDI) [44] were calculated to validate the added predictive value of the miRNAs. All *p*-values were two-tailed and a level of *p* < 0.05 was considered statistically significant.

### 4.6. Ethics Statement

The protocol of this study was carried out according to the principles of the Declaration of Helsinki and approved by the Medical Ethics Committee of the First Affiliated Hospital of Zhengzhou University. Written informed consent was obtained from all the participants before enrolment.

## 5. Conclusions

LV remodelling occurs in quite a large number of AMI patients and is associated with increased risk of cardiovascular death and the progression of heart failure, therefore early identification of this consequence of AMI is clinically very important. Results of our study revealed that plasma miR-208b and miR-34a could serve as available predictors for LV remodeling after AMI and were associated with the six months mortality or development of heart failure. Despite a modest prognostic power, our present study lays the groundwork for future efforts to identify and develop miR-208b and miR-34a (perhaps also other miRNAs) as novel class of blood-based biomarkers for AMI prognosis.

## Figures and Tables

**Table 1.** Demographic, clinical features, laboratory data and echo parameters.

Characteristics	Total patients (*n* = 359)	Remodeling (*n* = 116)	Non-remodeling (*n* = 243)	*p*_1_	Experienced endpoint (*n* = 83)	No endpoint (*n* = 276)	*p*_2_
Age (years)	58 ± 14	59 ± 12	57 ± 15	0.587	57 ± 11	58 ± 14	0.805
Male/female (*n*/*n*)	301/58	97/19	204/39	0.937	75/8	226/50	0.066
Current smoking, *n* (%)	190 (53%)	64 (55%)	124 (51%)	0.462	44 (53%)	146 (53%)	0.986
Diabetes mellitus, *n* (%)	57 (16%)	22 (19%)	35 (14%)	0.269	15 (18%)	42 (15%)	0.533
Hypertension, *n* (%)	172 (48%)	60 (52%)	109 (45%)	0.223	33 (40%)	139 (50%)	0.090
Hyperlipidaemia, *n* (%)	126 (35%)	44 (38%)	78 (32%)	0.275	32 (38%)	94 (34%)	0.452
SBP (mmHg)	123 ± 21	127 ± 25	120 ± 16	0.155	122 ± 28	124 ± 18	0.824
DBP (mmHg)	76 ± 12	77 ± 9	74 ± 12	0.949	74 ± 15	77 ± 12	0.360
TC (mmol/L)	3.99 ± 1.07	3.80 ± 0.94	4.16 ± 1.15	0.112	3.73 ± 1.15	4.07 ± 1.04	0.199
TG (mmol/L)	1.55 ± 0.91	1.61 ± 0.98	1.50 ± 0.85	0.564	1.58 ± 1.01	1.54 ± 0.89	0.859
HDL (mmol/L)	1.03 ± 0.30	1.00 ± 0.26	1.07 ± 0.32	0.289	0.95 ± 0.24	1.07 ± 0.31	0.125
LDL (mmol/L)	2.41 ± 0.82	2.23 ± 0.57	2.43 ± 0.97	0.093	2.15 ± 0.70	2.49 ± 0.84	0.089

AMI onset to sample (h; median(range))	6 (2–10)	6 (2–10)	6 (2–10)	0.473	6 (3–10)	6 (2–10)	0.293

discharge to follow up (days; median(range))	176 (121–226)	170 (121–214)	182 (133–226)	0.248	179 (134–214)	172 (121–226)	0.322

Serum biomarkers during admission (median(IQR))							

Peak CK (U/L)	1536 (239,6839)	1474 (191,5805)	1609 (286,7017)	0.119	1616 (253,6378)	1390 (193,7082)	0.094
Cardiac troponin T (ng/mL)	12.33 (0.088,53.32)	15.65 (0.35,58.44)	10.65 (0.004,47.21)	0.015	13.13 (0.54,63.46)	12.07 (0.005,49.57)	0.075
Nt-pro-BNP (pg/mL)	350 (145,807)	507 (212,1057)	279 (81,733)	0.003	567 (253,1189)	233 (116,773)	0.001

Medications, *n* (%)							

Beta-blockers	305 (85%)	100 (86%)	205 (84%)	0.674	67 (81%)	238 (86%)	0.218
Calcium antagonists	118 (33%)	44 (38%)	74 (30%)	0.158	33 (40%)	85 (31%)	0.128
ACEI/ARB	219 (61%)	66 (57%)	153 (63%)	0.270	44 (53%)	175 (63%)	0.089
Statins	352 (98%)	114 (98%)	238 (98%)	0.831 a	83 (100%)	269 (97%)	0.143 a
Anti-platelet therapy	359 (100%)	116 (100%)	243 (100%)	1.000	83 (100%)	276 (100%)	1.000
Diuretic	126 (35%)	47 (41%)	79 (33%)	0.137	35 (42%)	91 (33%)	0.124

Treatment, *n* (%)							

CAG	291 (81%)	93 (80%)	198 (81%)	0.767	68 (82%)	223 (81%)	0.818
Thrombolysis	183 (51%)	60 (52%)	123 (51%)	0.844	41 (49%)	142 (51%)	0.743
PCI	244 (68%)	74 (64%)	170 (70%)	0.242	55 (66%)	189 (68%)	0.705

Pre-discharge echo (median(IQR))							

LVEDV (mL)	108 (97,119)	108 (89,119)	108 (103,119)	0.595	106 (89,121)	109 (98,119)	0.334
LVESV (mL)	47 (37,54)	48 (37,56)	47 (38,53)	0.489	45 (37,54)	47 (38,54)	0.772
LVEF (%)	60 (56,64)	60 (56,64)	60 (56,63)	0.412	60 (56,66)	60 (56,63)	0.394

Follow-up echo (median(IQR))							

LVEDV (mL)	120 (102,131)	124 (110,132)	112 (100,122)	0.034	121 (104,132)	120 (102,130)	0.850
LVESV (mL)	49 (41,55)	48 (38,55)	49 (45,55)	0.427	51 (44,57)	48 (39,55)	0.366
LVEF (%)	55 (49,61)	51 (47,58)	59 (55,63)	0.027	54 (48,62)	56 (52,60)	0.575

Change between discharge and follow-up (median(IQR))							

ΔLVEDV (mL)	10 (2,18)	18 (14,24)	3 (−4,5)	0.000	17 (13,26)	5 (−3,15)	0.000
ΔLVESV (mL)	2 (−1,4)	4 (1,6)	1 (−2,4)	0.005	4 (2,7)	2 (−1,4)	0.022
ΔLVEF (%)	−6 (−8,2)	−9 (−13,2)	1 (−4,4)	0.000	−6 (−11,2)	−1 (−3,3)	0.003

*p*_1_: Comparison between remodeling and non-remodeling group; *p*_2_: Comparison between experienced endpoint and no endpoint group.

a By continuity correction chi-square test.

IQR: Interquartile range; SBP: systolic blood pressure; DBP: Diastolic blood pressure; TC: Total cholesterol; TG: Triglyceride; HDL: High-density lipoprotein; LDL: Low-density lipoprotein; CK: Creatine kinase; NT-proBNP: *N*-terminal pro-brain natriuretic peptide; ACEI: Angiotensin-converting enzyme inhibitor; ARB: Angiotensin II receptor blocker; CAG: Coronary arteriography; PCI: Percutaneous Coronary Intervention; LVEDV: Left ventricular end-diastolic volume; LVESV: Left ventricular end-systolic volume; LVEF: left ventricular ejection fraction.

**Table 2.** MiR-208b and miR-34a in each group.

MiRNAs	Δ*C*_t_/ΔΔ*C*_t_	Remodeling (*n* = 116)	Non-remodeling (*n* = 243)	*p*_1_	Experienced endpoint (*n* = 83)	No endpoint (*n* = 276)	*p*_2_
miR-208b	Δ*C*_t_	2.86 ± 1.30	4.04 ± 1.61	0.000	2.47 ± 1.48	3.50 ± 1.39	0.004
	ΔΔ*C*_t_	−1.94 ± 1.27	0		−1.35 ± 1.48	0	
miR-34a	Δ*C*_t_	3.06 ± 1.12	4.06 ± 1.59	0.001	2.93 ± 1.63	3.77 ± 1.54	0.035
	ΔΔ*C*_t_	−1.32 ± 1.12	0		−0.94 ± 1.63	0	

*p*_1_: Comparison between remodeling and non-remodeling group; *p*_2_: Comparison between experienced endpoint and no endpoint group. Δ*C*_t_ and ΔΔ*C*_t_ value of miR-208b and miR-34a in each group is presented as an average group Δ*C*_t_ ± SD. Corresponding *p* values were calculated using the Independent-samples *T* test.

**Table 3.** NRI analysis for miR-208b.

Model a without miR-208b	Model a with miR-208b				Reclassification			
Predicted risk	<10%	10%–30%	>30%	Total	Increased risk, *n* (%)	Decreased risk, *n* (%)	NRI b	*p*

Patients with remodeling (*n* = 116)								

<10%	27	9	5	41				
10%–30%	4	35	4	43				
>30%	0	3	29	32				
Total	31	47	38	116	18 (15.5)	7 (6.0)		

Patients without remodeling (*n* = 243)								

<10%	98	3	2	103				
10%–30%	5	99	3	107				
>30%	0	3	30	33				
Total	103	105	35	243	8 (3.3)	8 (3.3)		
NRI b							0.095	0.039

Patients were categorized into <10%, 10%–30% and >30% probability of remodeling.

a Multi-parameter clinical model included age, gender, current smoking, cTnT, NT-proBNP, and time from AMI onset to sampling;

b NRI = [*p* (up|D = 1) − *p* (down|D = 1)] − [*p* (up|D = 0) − *p* (down|D = 0)].

**Table 4.** NRI analysis for miR-34a.

Model a without miR-34a	Model a with miR-34a				Reclassification			
Predicted risk	<10%	10%–30%	>30%	Total	Increased risk, *n* (%)	Decreased risk, *n* (%)	NRI b	*p*

Patients with remodeling (*n* = 116)								

<10%	26	7	3	36				
10%–30%	5	35	7	47				
>30%	0	4	29	33				
Total	31	46	39	116	17 (14.7)	9 (7.8)		

Patients without remodeling (*n* = 243)								

<10%	93	4	3	100				
10%–30%	4	97	5	106				
>30%	0	7	30	37				
Total	97	108	38	243	12 (4.9)	11 (4.5)		
NRI b							0.065	0.177

Patients were categorized into <10%, 10%–30% and >30% probability of remodeling.

a Multi-parameter clinical model included age, gender, current smoking, cTnT, NT-proBNP, and time from AMI onset to sampling;

b NRI = [*p* (up|D = 1) − *p* (down|D = 1)] − [*p* (up|D = 0) − *p* (down|D = 0)].

**Table 5.** NRI analysis for miR-208b and miR-34a.

Model a without miR-208b and miR-34a	Model a with miR-208b and miR-34a				Reclassification			
Predicted risk	<10%	10%–30%	>30%	Total	Increased risk, *n* (%)	Decreased risk, *n* (%)	NRI b	*p*

Patients with remodeling (*n* = 116)								

<10%	25	11	3	39				
10%–30%	4	35	8	47				
>30%	0	4	26	30				
Total	29	50	37	116	22 (19.0)	8 (6.9)		

Patients without remodeling (*n* = 243)								

<10%	89	6	3	98				
10%–30%	8	95	6	109				
>30%	0	6	30	36				
Total	97	107	39	243	15 (6.2)	14 (5.8)		
NRI b							0.117	0.025

Patients were categorized into <10%, 10%–30% and >30% probability of remodeling.

a Multi-parameter clinical model included age, gender, current smoking, cTnT, NT-proBNP, and time from AMI onset to sampling;

b NRI = [*p* (up|D = 1) − *p* (down|D = 1)] − [*p* (up|D = 0) − *p* (down|D = 0)].
